# Response: Commentary: Effects of dividing attention on memory for declarative and procedural aspects of tool use

**DOI:** 10.3389/fpsyg.2018.00631

**Published:** 2018-05-03

**Authors:** Shumita Roy, Norman W. Park

**Affiliations:** Psychology, York University, Toronto, ON, Canada

**Keywords:** tool use, motor skill learning, declarative memory, procedural memory, technical reasoning

Osiurak et al. (2016) presented a commentary on our previous research. However, we found that their commentary contained several inaccurate statements and misrepresented the purpose and implications of our work. Thus, we will begin by describing our research objectives and findings. We will then offer a critique of the position taken by Osiurak et al. regarding the processes of tool use. Finally, we will discuss how our research contributes to a better understanding of the processes involved in skilled tool use.

## Research objectives of our novel tool use paradigm

According to Osiurak et al. (2016; p. 2), we proposed that “tool use is mainly based on declarative and procedural aspects.” This statement does not exist in any of our published work. Thus, we wish to clarify that a primary purpose of our previous research was to study the acquisition and retention of tool use in order to investigate the relation between declarative and procedural memory systems. It was never our objective to specify fully all of the cognitive processes involved in tool use. We have studied the role of different memory systems in the acquisition of tool-related knowledge and skills by examining patients with isolated deficits in one of these memory systems (e.g., amnesia, Parkinson's disease), as well as healthy participants (Roy and Park, 2010, 2016; Roy et al., 2015; Fernandes et al., 2017). Based on our findings, we have concluded that memory for tool attributes (e.g., function, physical features) relies heavily on declarative memory, or conceptual knowledge. In contrast, skill acquisition and retention rely predominantly on motor procedural memory, but that there may be some instances where conceptual knowledge is also involved in skill learning. Lastly, we concluded that skilled tool use may rely on an interaction of both declarative tool knowledge and motor procedural skills.

## Tool use is not only about technical reasoning

We now turn to a critique of Osiurak et al.'s (2016; p. 2) thesis that, “the core aspect of tool use may be technical reasoning.” Despite accusing us of attributing tool use primarily to the realm of memory, Osiurak et al. fall prey to their own critique by attributing tool use primarily to technical reasoning. We acknowledge that in cases where the operation and goal of the tool may be relatively transparent, technical reasoning may be highly effective in achieving accurate tool use. Work with nonhuman primates has shown evidence of this (Proffitt et al., 2016). However, as discussed subsequently, with many types of complex tools, technical reasoning may not be sufficient.

Osiurak et al. (2016; p. 1) state that the idea of tool use relying on a cooperation of memory systems is “at odds with recent theoretical and empirical advances.” Yet, they fail to consider an entire body of literature highlighting the role of memory in tool use. Silveri and Ciccarelli (2009) observed that both physical tool use and functional knowledge about tools was degraded with severe semantic memory impairment. They further suggest that non-declarative forms of memory may compensate for semantic memory deficits. Baumard et al. (2016) in collaboration with the Osiurak group, found similar results, but they attributed compensation to technical reasoning rather than non-declarative aspects of memory. A dual-route hypothesis, involving some combination of action and knowledge, has been supported by others as well (Buxbaum et al., 1997; Rumiati and Humphreys, 1998; Buxbaum, 2017). However, what remains unclear is what the action component represents. Is it referring to general sensorimotor processes or striatally-mediated learned motor patterns, or both? Thus, we would argue that the technical reasoning hypothesis falls short in explaining all tool use and that other processes, such as memory, should be considered.

## Implications of our findings for tool use

A core assumption of Osiurak et al. (2016) is that participants derive the use of a tool through technical reasoning. Yet, for many tools, the function, manner of grasp, and manner of manipulation is unclear (see Buxbaum, 2017). For example, tools used by the trades (e.g., blowtorches, winches, belt sanders) come with a user's manual and people in these professions undergo training to learn how to use such tools. In a recent article, Osiurak and Badets (2016) proposed that certain tools such as phones and computers, are mediated by procedural learning since mechanical knowledge cannot support their use. Findings from our studies are partly consistent with this hypothesis. In our studies, we deliberately designed novel tools in which the function and use of the tools could not be determined via technical reasoning, as evidenced by an inability to accurately use the tool, or describe its function, prior to demonstration of correct use (Roy and Park, 2010; Fernandes et al., 2017). However, we concluded that both declarative and procedural memory played a critical role in tool use.

A further limitation of the technical reasoning perspective is that it does not account for increased speed and proficiency with which people tend to use tools with extended practice. Our research focuses on understanding the cognitive and neural processes that mediate this improved performance with practice. We have shown that, healthy participants become faster in performing tool tasks and retain more details about tool attributes with practice (Roy and Park, 2016). Findings from our patient studies further suggest that improved ability to use tools with practice, is mediated by two distinct memory systems, which retain different aspects of tool use and knowledge (Roy and Park, 2010; Fernandes et al., 2017). Osiurak and Badets (2016) proposed that expertise is acquired through progressive acquisition of mechanical knowledge. However, this implies that there is a learned component associated with prolonged tool use. We would argue that declarative and procedural memory systems are critical in supporting these learned aspects of tool use.

## Conclusion

We do not find our research to be at odds with that of Osiurak and colleagues. Rather, we view our research programs as complementary. The intersection of memory and tool use may lead to valuable insights about memory, but also help to clarify the cognitive processes supporting tool use.

## Author contributions

Both SR and NP have made significant and equal intellectual contribution to the production of this manuscript and have approved the final version for publication.

### Conflict of interest statement

The authors declare that the research was conducted in the absence of any commercial or financial relationships that could be construed as a potential conflict of interest.
